# *Ralstonia solanacearum* type III effector RipAS associates with potato type one protein phosphatase StTOPP6 to promote bacterial wilt

**DOI:** 10.1093/hr/uhad087

**Published:** 2023-05-03

**Authors:** Bingsen Wang, Wenfeng He, Mengshu Huang, Jiachen Feng, Yanping Li, Liu Yu, Yuqi Wang, Dan Zhou, Chengzhen Meng, Dong Cheng, Ning Tang, Botao Song, Huilan Chen

**Affiliations:** National Key Laboratory for Germplasm Innovation & Utilization of Horticultural Crops, Huazhong Agricultural University, Wuhan 430070, China; Key Laboratory of Potato Biology and Biotechnology (HZAU), Ministry of Agriculture and Rural Affairs, Wuhan 430070, China; Potato Engineering and Technology Research Center of Hubei Province, Huazhong Agricultural University, Wuhan 430070, China; College of Horticulture and Forestry Science, Huazhong Agricultural University, Wuhan 430070, China; National Key Laboratory for Germplasm Innovation & Utilization of Horticultural Crops, Huazhong Agricultural University, Wuhan 430070, China; Key Laboratory of Potato Biology and Biotechnology (HZAU), Ministry of Agriculture and Rural Affairs, Wuhan 430070, China; Potato Engineering and Technology Research Center of Hubei Province, Huazhong Agricultural University, Wuhan 430070, China; College of Horticulture and Forestry Science, Huazhong Agricultural University, Wuhan 430070, China; National Key Laboratory for Germplasm Innovation & Utilization of Horticultural Crops, Huazhong Agricultural University, Wuhan 430070, China; Key Laboratory of Potato Biology and Biotechnology (HZAU), Ministry of Agriculture and Rural Affairs, Wuhan 430070, China; Potato Engineering and Technology Research Center of Hubei Province, Huazhong Agricultural University, Wuhan 430070, China; College of Horticulture and Forestry Science, Huazhong Agricultural University, Wuhan 430070, China; State Key Laboratory of Crop Stress Adaptation and Improvement, Henan University, Kaifeng 475001, China; National Key Laboratory for Germplasm Innovation & Utilization of Horticultural Crops, Huazhong Agricultural University, Wuhan 430070, China; Key Laboratory of Potato Biology and Biotechnology (HZAU), Ministry of Agriculture and Rural Affairs, Wuhan 430070, China; Potato Engineering and Technology Research Center of Hubei Province, Huazhong Agricultural University, Wuhan 430070, China; College of Horticulture and Forestry Science, Huazhong Agricultural University, Wuhan 430070, China; National Key Laboratory for Germplasm Innovation & Utilization of Horticultural Crops, Huazhong Agricultural University, Wuhan 430070, China; Key Laboratory of Potato Biology and Biotechnology (HZAU), Ministry of Agriculture and Rural Affairs, Wuhan 430070, China; Potato Engineering and Technology Research Center of Hubei Province, Huazhong Agricultural University, Wuhan 430070, China; College of Horticulture and Forestry Science, Huazhong Agricultural University, Wuhan 430070, China; National Key Laboratory for Germplasm Innovation & Utilization of Horticultural Crops, Huazhong Agricultural University, Wuhan 430070, China; Key Laboratory of Potato Biology and Biotechnology (HZAU), Ministry of Agriculture and Rural Affairs, Wuhan 430070, China; Potato Engineering and Technology Research Center of Hubei Province, Huazhong Agricultural University, Wuhan 430070, China; College of Horticulture and Forestry Science, Huazhong Agricultural University, Wuhan 430070, China; National Key Laboratory for Germplasm Innovation & Utilization of Horticultural Crops, Huazhong Agricultural University, Wuhan 430070, China; Key Laboratory of Potato Biology and Biotechnology (HZAU), Ministry of Agriculture and Rural Affairs, Wuhan 430070, China; Potato Engineering and Technology Research Center of Hubei Province, Huazhong Agricultural University, Wuhan 430070, China; College of Horticulture and Forestry Science, Huazhong Agricultural University, Wuhan 430070, China; National Key Laboratory for Germplasm Innovation & Utilization of Horticultural Crops, Huazhong Agricultural University, Wuhan 430070, China; Key Laboratory of Potato Biology and Biotechnology (HZAU), Ministry of Agriculture and Rural Affairs, Wuhan 430070, China; Potato Engineering and Technology Research Center of Hubei Province, Huazhong Agricultural University, Wuhan 430070, China; College of Horticulture and Forestry Science, Huazhong Agricultural University, Wuhan 430070, China; National Key Laboratory for Germplasm Innovation & Utilization of Horticultural Crops, Huazhong Agricultural University, Wuhan 430070, China; Key Laboratory of Potato Biology and Biotechnology (HZAU), Ministry of Agriculture and Rural Affairs, Wuhan 430070, China; Potato Engineering and Technology Research Center of Hubei Province, Huazhong Agricultural University, Wuhan 430070, China; College of Horticulture and Forestry Science, Huazhong Agricultural University, Wuhan 430070, China; State Key Laboratory of Crop Stress Adaptation and Improvement, Henan University, Kaifeng 475001, China; National Key Laboratory for Germplasm Innovation & Utilization of Horticultural Crops, Huazhong Agricultural University, Wuhan 430070, China; Key Laboratory of Potato Biology and Biotechnology (HZAU), Ministry of Agriculture and Rural Affairs, Wuhan 430070, China; Potato Engineering and Technology Research Center of Hubei Province, Huazhong Agricultural University, Wuhan 430070, China; College of Horticulture and Forestry Science, Huazhong Agricultural University, Wuhan 430070, China; National Key Laboratory for Germplasm Innovation & Utilization of Horticultural Crops, Huazhong Agricultural University, Wuhan 430070, China; Key Laboratory of Potato Biology and Biotechnology (HZAU), Ministry of Agriculture and Rural Affairs, Wuhan 430070, China; Potato Engineering and Technology Research Center of Hubei Province, Huazhong Agricultural University, Wuhan 430070, China; College of Horticulture and Forestry Science, Huazhong Agricultural University, Wuhan 430070, China

## Abstract

The bacterial pathogen *Ralstonia solanacearum* (*R. solanacearum*) delivered type III secretion effectors to inhibit the immune system and cause bacterial wilt on potato. Protein phosphatases are key regulators in plant immunity, which pathogens can manipulate to alter host processes. Here, we show that a type III effector RipAS can reduce the nucleolar accumulation of a type one protein phosphatase (PP1) StTOPP6 to promote bacterial wilt. StTOPP6 was used as bait in the Yeast two-Hybrid (Y2H) assay and acquired an effector RipAS that interacts with it. RipAS was characterized as a virulence effector to contribute to *R. solanacearum* infection, and stable expression of RipAS in potato impaired plant resistance against *R. solanacearum.* Overexpression of StTOPP6 showed enhanced disease symptoms when inoculated with wild strain UW551 but not the *ripAS* deletion mutant, indicating that the expression of StTOPP6 facilitates the virulence of RipAS. RipAS reduced the nucleolar accumulation of StTOPP6, which occurred during *R. solanacearum* infection. Moreover, the association also widely existed between other PP1s and RipAS. We argue that RipAS is a virulence effector associated with PP1s to promote bacterial wilt.

## Introduction

Bacterial wilt is the most serious bacterial disease affecting potato production and leads to great economic losses worldwide each year [1]. The *Ralstonia solanacearum* (*R. solanacearum*) has a wide host range, while phylotype IIB is the main pathogen infecting potato with global distribution [2]. Both potato and phylotype IIB *R. solanacearum* originated in South America, indicating that they may have a long history of co-evolution [3]. Therefore, it is a common strategy to tap potential resistance resources through the interaction of *R. solanacearum* and potato.

T3Es are the core virulence factors of *R. solanacearum* and can be transferred into host cells through the type III secretion system (T3SS), a ‘molecular syringe’ to modulate host defenses [4, 5]. With a large number of effectors, *R. solanacearum* can adapt to different hosts, and contribute to infecting more than 200 different plant species [6, 7]. Due to the long coevolutionary relationship between effectors and plant immunity, effectors can be used to detect the key proteins of plant immunity.

As a model pathogen for root and vascular diseases, *R. solanacearum* are pathogenic bacteria with a great quantity of functionally characterized T3Es. A pan-effectome of 140 *R. solanacearum* strains was generated, including 102 T3Es and 16 hypothetical T3Es [8]. Statistically, more than 50 different effectors have been characterized to varying degrees [9]. Disruption of the plant defense is the main studied function of pathogenic effectors, such as RipAB disrupts SA signaling by targeting TGA transcription factors to suppress plant immunity [10], and RipAC prevents MAPK-mediated phosphorylation of SGT1 to suppress plant immunity [11]. However, there are still a large number of effectors’ functional characteristics that remain unknown. One of the main reasons for the complexity of effector studies is the discovery of genetic redundancy in different T3Es, and while this can ensure greater pathogenicity of bacteria, the functional dissection of individual effectors becomes more complex, especially for homologous families [12]. The T3Es have evolved sufficiently to adapt to the potato immune system over a long period of natural evolution, and can be used as an indispensable molecular probe for studying potato immunity.

Protein phosphatase 1 (PP1), also known as type one protein phosphatase (TOPP), expressed in all eukaryotic cells and accounts for main cellular Ser/Thr dephosphorylation, and nine TOPPs have been identified in Arabidopsis [13, 14]. Not only are the sequence and structure of PP1 highly conserved but also it is involved in regulating cellular processes, including mRNA transcription, protein translation, and metabolism [15–17].

Protein phosphatase has been reported to play a critical role in plant biotic stress response [18]. For instance, protein phosphatase 2C38 modulates the phosphorylation and activation status of BIK1 to negatively regulate the defense response [19]. Protein phosphatase 2A targets BAK1 to suppress PAMP-triggered responses [20]. In Arabidopsis, TOPPs directly interacted with MAPKs and affected the MAPK-mediated signaling pathway to regulate plant immunity [21]. Numerous studies have shown that pathogenic bacteria can target protein phosphatases to interfere with plant immunity, or manipulate plant protein phosphatases to enhance virulence. Pathogens can promote PP2C-type phosphatase HAI1 in host cells to suppress the activation of MAPKs, and contribute to enhancing pathogen virulence [22]. Two AvrE-family T3Es WtsE and AvrE1 manipulate host immunity by targeting sub-component specific PP2A complexes [23]. The *Phytophthora infestans* effector Pi04314 increases the protein phosphatase activity by interacting with protein phosphatase 1 catalytic (PP1c) isoforms and forming holoenzyme to promote late blight in potato [24]. However, there is no report that effectors target or manipulate plant protein phosphatase to their advantage in *R. solanacearum.*

Previously, we found that the silencing of *NbPP1*, a homologous gene of type one protein phosphatase StTOPP6 (NCBI Reference Sequence: XM_006350625.2) in potato, increased resistance to *R. solanacearum* [25]*.* We argue that StTOPP6 is a susceptibility factor to promote bacterial wilt. In this study, to screen for effectors potentially associated with StTOPP6, we constructed a yeast library containing 56 T3Es of *R. solanacearum* UW551. The T3Es RipAS was obtained by screening with StTOPP6 as a decoy protein and further confirmed the interaction between the RipAS and StTOPP6. RipAS is identified as a virulence effector by Δ*ripAS* mutant and potato transgenic lines and acts to enhance bacterial wilt disease. Furthermore, the presence of StTOPP6 facilitated the infection of WT *R. solanacearum* UW551, but not Δ*ripAS* mutant. We propose a model whereby RipAS manipulate StTOPP6 by interacting with StTOPP6 and reducing its nucleolar accumulation to promote bacterial wilt.

## Results

### RipAS associates with StTOPP6 in potato

We previously identified a type one protein phosphatase StTOPP6 in potato, which negatively regulated the bacterial wilt resistance [25]. To confirm the presence of effectors targeting StTOPP6 in *R. solanacearum*, 52 effectors were cloned in the vector pGADT7 (Fig. S1A, see online supplementary material). Yeast growth state results show that the size of the effectors did not affect the transformation efficiency (Fig. S1B, see online supplementary material). Finally mixing the effectors plasmids in equal proportions, a yeast library containing all identified effectors of the *R. solanacearum* strain UW551 was obtained. StTOPP6 was used as a bait to screen potential effectors by Yeast two-Hybrid (Y2H) assay, sequencing of the blue single clones results all matched the effector RipAS (Fig. S1C, see online supplementary material).

**Figure 1:**
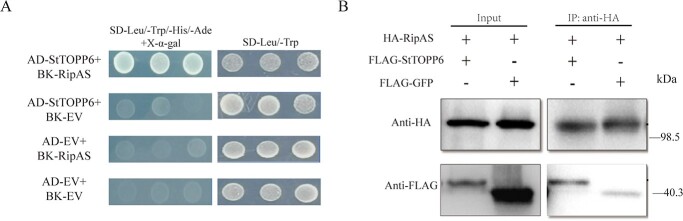
RipAS physically interacted with StTOPP6. **A** RipAS interacts with StTOPP6 in Y2H. The combinations of AD-StTOPP6 and BD-EV or BK-RipAS and AD-EV were negative controls. **B** RipAS interacts with StTOPP6 in the Co-IP assay. Total protein was extracted to immunoprecipitate with HA-tag beads and immunoblotted with anti-FLAG antibody.

To reconfirm interactions, pairwise Y2H was performed by exchanging the yeast expression vectors between StTOPP6 and RipAS, RipAS interacted with StTOPP6 as indicated by growth on quadruple drop-out selection media and induction of α-galactosidase activity, whereas the negative control did not (Fig. 1A). Then, to determine these interactions *in vivo*, the co-immunoprecipitation (Co-IP) assays were performed using HA-RipAS and FLAG-StTOPP6 constructs. The results showed that FLAG-StTOPP6 could be detected from the immunoprecipitated proteins of the HA-RipAS (Fig. 1B), indicating that RipAS interacted with StTOPP6 *in vivo*. To further investigated their association, Y2H and Co-IP assay were used to test the interaction between RipAS and StTOPP6m (the phosphatase-dead mutant of StTOPP6: His121Ala) [24] and found that RipAS could not interact with StTOPP6m (Fig. S1D and E, see online supplementary material). Altogether, these results suggest that RipAS can interact with StTOPP6, and possibly be associated with phosphatase activity.

### RipAS contributes to *R. solanacearum* infection

RipAS is identified as a type III effector according to the uniform nomenclature (https://iant.toulouse.inra.fr/T3E) of *R. solanacearum* T3Es [26]. To test the secretion of RipAS, an adenylate cyclase (cya) assay was performed [27]. The fusion protein RipAS-Cya was promoted by the native promoter and transformed into UW551, while the T3SS mutant Δ*hrcV* as a negative control. *R. solanacearum* strains were inoculated into potato tubers, and the concentration of cAMP in plants inoculated with UW551 (RipAS-Cya) was significantly higher than that in plants inoculated with the other test strains (Fig. S2A, see online supplementary material), indicating that RipAS is a type III secretion effector.

**Figure 2:**
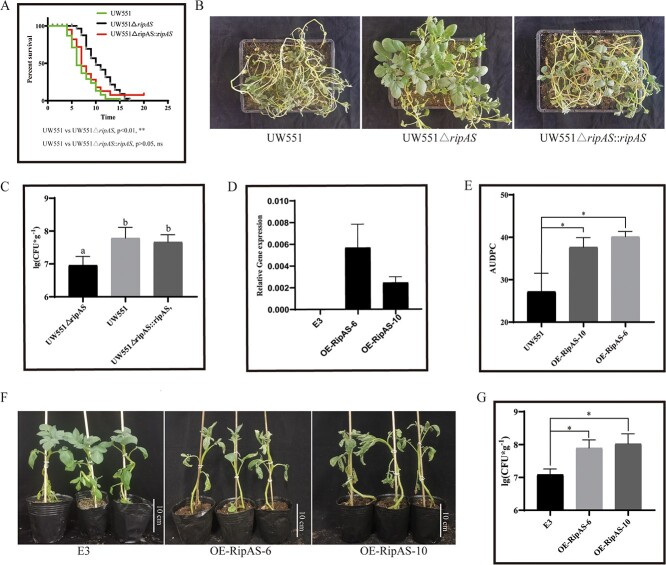
RipAS is required for full virulence in potato. **A** Survival analysis to compare the virulence of *Ralstonia solanacearum* strain UW551, a *ripAS* mutant, and the complementation strain Δ*ripAS*::*ripAS*. The survival rates showed one independent experiment, each experiment containing 48 plants (GehanBreslow-Wilcoxon test, **P < 0.01, ns *P* > 0.05). **B** Disease symptoms of potato inoculated with *R. solanacearum* strains at 14 days dpi. **C***In vivo* the bacterial populations of potato roots at 7 dpi. **D** The expression of *ripAS* in OE-RipAS lines and the control plants E3. Relative transcript levels were normalized to the *Stef1α*. **E** The AUDPC values of OE-RipAS lines and WT E3 plants inoculated with *R. solanacearum* strain UW551. The mean of the AUDPC value was assessed from 0 to 21 dpi. **F** Representative photographs showing the disease symptoms of OE-RipAS lines at 10 dpi. **G***In vivo* the bacterial populations of OE-RipAS lines and WT E3 plants at 7 dpi. In (**C**, **E**, **G**), Error bars represent standard errors and an asterisk (*) = *P* < 0.05 by Dunnett’s multiple comparison test. (** indicates *P* < 0.01, ns indicates *P* ≥ 0.05). dpi means days postinoculation. Three independent biological replicates were performed with similar results.

To identify the involvement of RipAS on UW551 virulence, a Δ*ripAS* deleting mutant in the UW551 strain was generated (Fig. S2B, see online supplementary material), and the growth state of this mutant is unaffected (Fig. S2C, see online supplementary material). However, the contribution of individual T3Es to the natural infection process is often difficult to determine, considering functional redundancy among them, inoculated bacteria were drenched in a pot containing 48 (6 × 8) potato plants, and the surviving plants were recorded. The potato infection assays showed an attenuated disease symptom inoculated with Δ*ripAS* mutant. Genetic complementation of Δ*ripAS* mutant strain Δ*ripAS*::*ripAS* was constructed, and potato infection results showed the complementation strain recovered the virulence of Δ*ripAS* mutant to WT level (Fig. 2A and B). These results indicated that the attenuated virulence was indeed due to the lack of the *ripAS*. A quantitative analysis of pathogen colonization was performed to confirm the differences in root bacterial colonization levels. Consistent with potato infection results, the decreased growth of Δ*ripAS* mutant was detected in potato roots (Fig. 2C), indicating that RipAS contribute to the *R. solanacearum* infection*.*

To give further insights into the virulence of RipAS, we generate transgenic lines to stably express RipAS by a 35S constitutive promoter in potato. Two independent potato transgenic lines (OE-RipAS-6 and OE-RipAS-10) were subsequently identified by qRT-PCR and SqRT-PCR (Fig. 2D; Fig. S3A, see online supplementary material). Independent transgenic lines expressing RipAS were challenged with *R. solanacearum* UW551 and showed enhanced disease symptoms, as measured by the AUDPC and bacterial colonization (Fig. 2E–G). Thus, these results confirm that RipAS impaired plant resistance against *R. solanacearum*.

Although the growth of transgenic lines was not otherwise perturbed (Fig. S3B, see online supplementary material), expressing RipAS in potato led to a lower tuber yield than that of the untransformed WT E3 control lines (Fig. S3C and D). Therefore, RipAS could also affect the development of potato. To identify plant processes affected by RipAS, RNA-seq analysis was then performed using non-inoculated roots of WT and two transgenic lines and eliminated outlier samples based on cluster analysis (Fig. S4A; Data S1, see online supplementary material). A total of 316 differentially expressed genes (DEGs, fold change >2, and *P*-value <0.05) were identified (Fig. S4B, see online supplementary material). Based on GO functional categorization, genes related to the defense response to stress, response to endogenous stimulus, and response to abiotic stress were significantly enriched (Fig. S4C, see online supplementary material), while development-related terms, such as photosynthesis, photosynthesis proteins, flavonoid biosynthetic, and energy metabolism were enriched according to the kyoto encyclopedia of genes and genomes (KEGG) pathway enrichment analysis (Fig. S4D, see online supplementary material). Altogether, these results suggest that RipAS not only plays a significant role in *R. solanacearum* infection but also affects potato development.

### RipAS requires the presence of StTOPP6 to enhance virulence

**Figure 3:**
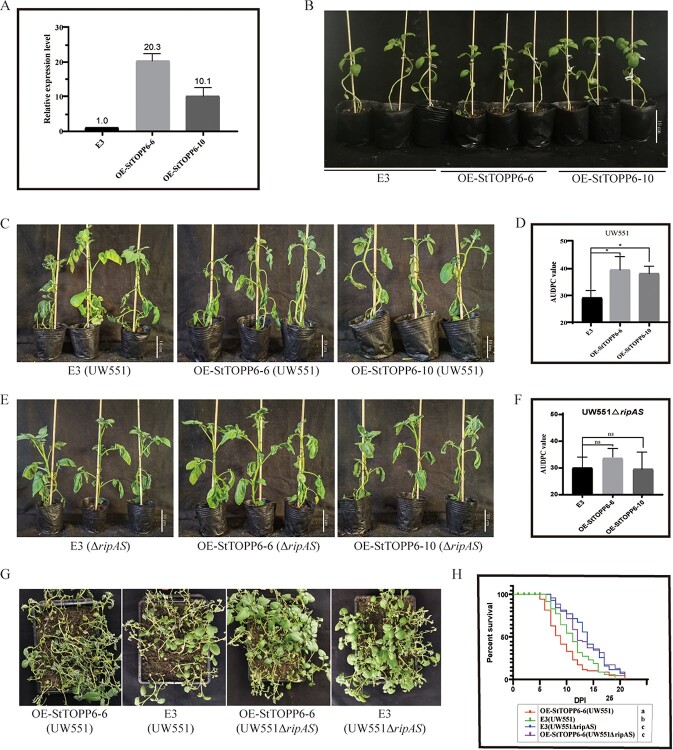
The presence of StTOPP6 results in increased virulence of RipAS. **A** qRT-PCR analyses of relative *StTOPP6* transcript levels in OE-StTOPP6 lines and the control plants E3. Relative transcript levels were normalized to the *Stef1α*. The expression of *StTOPP6* in the control plants was set to ‘1’. **B** Representative photos of OE-StTOPP6 lines and WT E3 plants grown in pots for 14 days in the greenhouse. **C** Disease symptoms of potato inoculated with UW551 stain by root irrigation at 10 dpi. **D** The AUDPC values of OE-StTOPP6 lines inoculated with *Ralstonia solanacearum* strain UW551. **E** Disease symptoms of potato inoculated with UW551Δ*ripAS* strains by root irrigation at 10 dpi. **F** The AUDPC values of OE-StTOPP6 lines inoculated with *R. solanacearum* strain UW551Δ*ripAS*. **G** Disease symptoms of potato inoculated with WT UW551 and Δ*ripAS* mutant strains by root irrigation at 14 days postinoculation (dpi). **H** Survival analysis of potato inoculated with WT UW551 and Δ*ripAS* mutant strains. The survival rates showed one independent experiment, each experiment containing 48 plants (GehanBreslow-Wilcoxon test, different lowercase letters indicate statistically significant differences (*P* < 0.05)). In (**D**, **F**), the mean of the AUDPC value was calculated from 0 to 21 dpi. Error bars represent standard errors (Dunnett’s multiple comparison test, ^*^P < 0.05, ns *P* ≥ 0.05). Three independent biological replicates were performed with similar results.

As the interaction experiments suggested that RipAS interacts with StTOPP6, we hypothesized StTOPP6 may be required for the full virulence of RipAS. GFP-fused StTOPP6 stable expression lines with StTOPP6 driven by 35S promoter were constructed. *StTOPP6* was highly expressed in two lines (OE-StTOPP6–6 and OE-StTOPP6–10), and the two lines were selected for further study (Fig. 3A and B). When inoculated with UW551, two transgenic lines displayed attenuated disease symptoms, as measured by the AUDPC and bacterial colonization (Fig. 3C and D), indicating that resistance attenuation was indeed caused by the overexpressing of StTOPP6. Whereas inoculated with Δ*ripAS* mutant strains, no significant difference in disease symptoms between OE-StTOPP6 lines and control E3 plants (Fig. 3E and F), which suggests that overexpression of STOPP6 did not affect potato resistance to bacterial wilt in the absence of RipAS. To further identify the difference in disease symptoms, OE-StTOPP6 lines and the control plant E3 were challenged with WT *R. solanacearum* UW551 and Δ*ripAS* mutant strains, separately and simultaneously. Although definite wilting symptoms were observed, there were significant differences in disease symptoms between the different combinations (Fig. 3G). OE-StTOPP6 lines inoculated with WT *R. solanacearum* UW551 showed significantly greater susceptibility, whereas the control plants E3 and OE-StTOPP6 lines inoculated with Δ*ripAS* mutant showed significantly greater resistance compared with the other combinations, as measured by the percent survival (Fig. 3H). These results further demonstrate that RipAS requires StTOPP6 to enhance the virulence of *R. solanacearum*.

### RipAS reduces the nucleolar accumulation of StTOPP6

**Figure 4:**
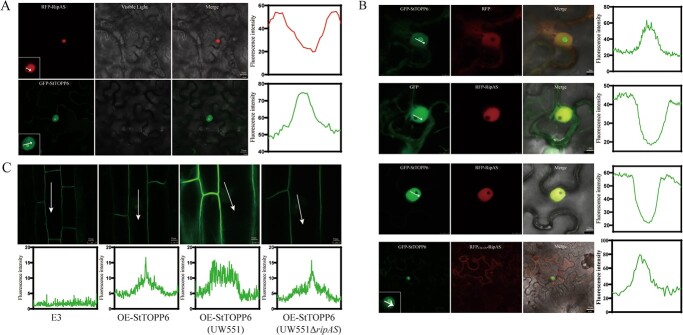
Co-expression of RipAS results in the reduced nucleolar accumulation of the StTOPP6. **A** The subcellular localization of StTOPP6 and RipAS. The scale bar is 10 μm. **B** The nucleolar fluorescence of GFP-StTOPP6 was reduced when co-expressed with RFP-RipAS. The scale bar is 5 μm. RFP_CBL6N_-RipAS decreases the accumulation in the nucleus, respectively. The scale bar is 20 μm. **C** The nucleolar fluorescence of GFP-StTOPP6 was reduced during *Ralstonia solanacearum* infection. Nucleolar GFP-StTOPP6 fluorescence was observed in root cells of OE-StTOPP6 lines. The scale bar is 5 μm. White arrows indicate peaks of GFP/RFP fluorescence intensity.

Fluorescence fusion proteins were generated (GFP–StTOPP6 and RFP–RipAS) to view the subcellular localization of the RipAS and StTOPP6 using confocal microscopy. StTOPP6 was observed to localize in the nucleoplasm, the nucleolus, and a little cytoplasmic, whereas the RipAS only expressed in the nucleoplasm (Fig. 4A). However, unlike the StTOPP6 protein, the phosphatase-dead StTOPP6m did not accumulate in the nucleolus (Fig. S5A, see online supplementary material).

Strikingly, the fluorescence of StTOPP6 displayed significantly reduced in nucleolar when co-expressed with RipAS, whereas there was no change when expressed with RFP (Fig. 4B). These results indicate that RipAS can alter the nucleolar localization of StTOPP6, and potentially dependent on its nucleoplasm localization. To verify this conjecture, we altered the subcellular localization of RipAS by fusing a tonoplast-localized signal peptide CBL6n. When co-localized with RFP_CBL6N_, the nucleolar fluorescence of GFP-StTOPP6 was unaffected (Fig. 4B). This indicated that the reduced nucleolar accumulation of StTOPP6 may be dependent on the nucleoplasm of RipAS localization.

The expression of StTOPP6 was detected in OE-RipAS lines and observed no significant difference (Fig. S5B, see online supplementary material). Co-expressed with RipAS or GFP showed there was no difference in StTOPP6 accumulation (Fig. S5C, see online supplementary material). These results indicated that RipAS did not perturb the expression of StTOPP6. In addition, the phosphatase activity level of StTOPP6 remained stable in the presence of RipAS (Fig. S5D, see online supplementary material). This means that RipAS cannot inhibit phosphatase activity.

To investigate the localization of StTOPP6 during infection, GFP-fused StTOPP6-overexpressing transgenic lines were inoculated with *R. solanacearum* UW551 and its Δ*ripAS* mutant strains. The nucleolar accumulation of StTOPP6 was significantly reduced in transgenic potato root cells infected with UW551, compared with infected and uninfected with Δ*ripAS* mutant (Fig. 4C). This indicates that the reduced nucleolar accumulation of StTOPP6 also occurs during *R. solanacearum* infection.

### RipAS interactions with PP1s are universal between *R. solanacearum* and hosts

**Figure 5:**
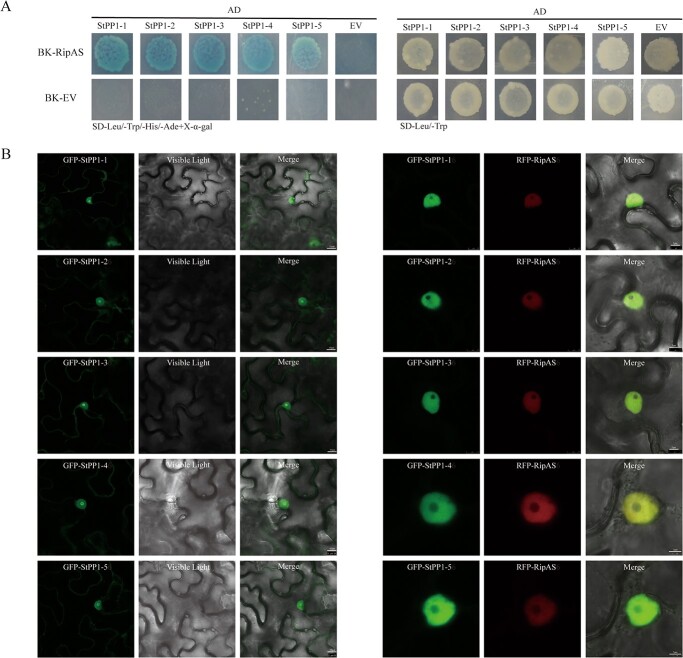
RipAS targets Type one Protein Phosphatases (PP1s) in potato. **A** Y2H assay to determine interactions between RipAS and StPP1s. The combinations of AD-StPP1s and BD-EV or BK-RipAS and AD-EV were negative controls. **B** The nucleolar fluorescence of GFP-StPP1s was reduced when co-expressed with RFP-RipAS. White arrows indicate peaks of GFP/RFP fluorescence intensity. The scale bar is 10 μm or 5 μm.

Type one protein phosphatase (TOPP) is the plant equivalent of PP1 phosphatase. PP1 is highly conserved among all eukaryotes, and the PP1 genes were homology searched by NCBI and Spud DB potato genomics resources. The other five homologous members of the PP1 gene family in potato were identified, named as StPP1–1 (XM_006354429.2), StPP1–2 (XM_006358645.2), StPP1–3 (XM_006350625.2), StPP1–4 (XM_006350729.2), and StPP1–5 (XM_006346756.2) (Fig. S6A, see online supplementary material). Associations of other PP1s with RipAS were also examined using the yeast system, and results showed that RipAS can also interact with StPP1–1, StPP1–2, StPP1–3, StPP1–4, and StPP1–5 (Fig. 5A). Therefore, we consider that the effector RipAS in *R. solanacearum* UW551 could interact with the PP1 family in potato. To further explore the associations of other family members with RipAS, each GFP fusion PP1 family gene was co-expressed with RFP–RipAS. Consistent with the subcellular of StTOPP6, other PP1 proteins displayed reduced fluorescence in the nucleolar when co-expressed with RFP-RipAS (Fig. 5B). These results suggest that not only StTOPP6 but also the PP1 proteins family is associated with RipAS.

**Figure 6:**
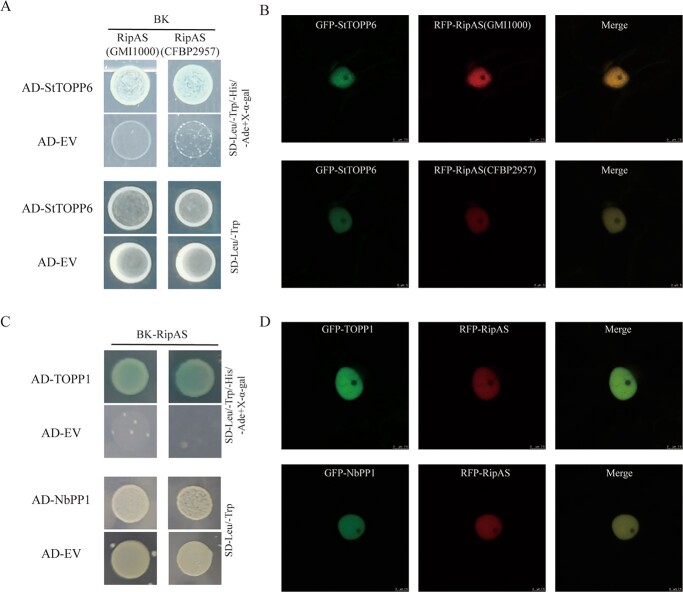
RipAS targets PP1s between *Ralstonia solanacearum* and hosts. **A** Y2H assay for interactions between RipAS in different *R. solanacearum* strains and StTOPP6. The combinations of AD-StTOPP6 and BD-EV or the other BK-RipAS and AD-EV were negative controls. **B** The nucleolar fluorescence of GFP-StTOPP6 was reduced when co-expressed with the other RFP-RipAS in *R. solanacearum*. The scale bar is 5 μm. **C** Y2H assay to determine the interactions between RipAS and PP1s in different hosts. The combinations of the other AD-PP1s and BD-EV or BK-RipAS and AD-EV were negative controls. **D** The nucleolar fluorescence of GFP-PP1s in different hosts was reduced when co-expressed with RFP-RipAS. White arrows indicate peaks of GFP/RFP fluorescence intensity. The scale bar is 7.5 μm.

Amino acid sequence alignment of RipAS shared high similarity in different phylotypes *R. solanacearum* (Fig. S6B, see online supplementary material). To investigate whether the StTOPP6 is also associated with the other RipAS, two RipAS (RipAS(GMI1000) and RipAS(CFBP2957)) were cloned and co-expressed with StTOPP6 in yeast and *Nicotiana benthamiana*. The interaction between RipAS and StTOPP6 has also occurred and the nucleolar GFP fluorescence of StTOPP6 was significantly reduced with the presence of the other RipAS (Fig. 6A and B). To investigate whether the PP1 proteins in other plants are also associated with RipAS, TOPP1 (AT2G29400) in Arabidopsis and NbStPP1 (Niben101Scf03064g08007.1) in *N. benthamiana* also performed the same validation and the same results were shown as expected (Fig. 6C and D). In summary, the association between RipAS and PP1 proteins may widely exist during *R. solanacearum* infection.

## Discussion

More than 100 T3Es have been identified in *R. solanacearum*, but the mechanism of interacting with plants has not been fully uncovered. One of the main reasons is that the target protein is difficult to screen. Protein phosphatases are important immune regulators in plants, which have been reported to be targeted by pathogens. In *P. infestans*, effector Pi04314 can target plant PP1c isoforms to promote late blight disease [24]. Previously, we identified a type one protein phosphatase StTOPP6 as a susceptibility factor. To verify the existence of *R. solanacearum* T3Es targeting StTOPP6, a yeast library containing all identified T3Es of UW551 was constructed. RipAS was successfully selected from this yeast library using StTOPP6 as bait, and the interaction between RipAS and StTOPP6 was confirmed (Fig. 1).

Firstly, we confirmed that the RipAS was secreted by T3SS during *R. solanacearum* infection (Fig. S2A, see online supplementary material). However, the function of RipAS has not been reported yet. Sequence analysis revealed that RipAS is an uncharacterized protein, but it is conserved in *R.solanacearum* species complex. T3E mutants can directly identify their contributions to virulence. However, the single effector knockout mutants often show the same phenotypes as the wild type due to the potential functional redundancy [27, 28]. We increased the number of inoculated plants and counted the survival rate to facilitate the identification of virulence differences, and successfully identified the virulence contribution of RipAS (Fig. 2A–C). Heterologous expression in plants is a powerful method to identify effector functions that interfere with plants immune system. For example, the transgenic ripAB-expressing potato plants show significant down-regulation of Ca^2+^ signals [29]. Here we showed that RipAS play an important role in the *R. solanacearum* infection process (Fig. 2). To understand the specific plant processes affected by RipAS, non-inoculated roots of RipAS-expression lines were subjected to conduct RNA-seq analysis. Defense-related GO terms were enriched in DEGs, indicating that the expression of RipAS can indeed interfere with plant defense (Fig. S4C, see online supplementary material). While development and growth-related terms were also enriched, the same results were obtained by KEGG pathway enrichment analysis (Fig. S4D, see online supplementary material). Consistent with the results of the RNA-seq analysis, expressing RipAS in potato have an effect on tuberization, including tuber number and yield (Fig. S3C and D). We believe that the virulence of RipAS not only interferes with the potato defense but also has an effect on the development of potato.

We found that overexpression of StTOPP6 displayed enhanced disease symptoms when inoculated with wild strain UW551, indicating that StTOPP6 is a susceptibility factor. However, when inoculated with *ripAS* deletion mutant, no different disease symptom was exhibited between StTOPP6 lines and E3 plants (Fig. 3), indicating that overexpression of StTOPP6 could not affect plant resistance against *ripAS*-deletion *R. solanacearum*. Therefore, we argue that the expression of StTOPP6 contributes to the virulence of RipAS during infection, and RipAS may target StTOPP6 to promote *R. solanacearum* infection.

StTOPP6 was observed to localize in the nucleus, including nucleoplasm and nucleolus, whereas RipAS was only expressed in the nucleoplasm. Markedly, GFP-StTOPP6 nucleolar fluorescence is reduced when co-expressed with RipAS (Fig. 4B). We have two hypotheses regarding the reduced StTOPP6 nuclear accumulation in the presence of RFP–RipAS. One is that RipAS causes the degradation of StTOPP6 in the nucleolus, which may result in reduced protein abundance and even altered phosphatase activity. In this study, we did not find evidence of differential StTOPP6 expression and phosphatase activity when co-expressed with RipAS or GFP (Fig. S5B–D, see online supplementary material). The other is that RipAS inhibits StTOPP6 from localizing to the nucleolus. StTOPP6 is known to be expressed in both the nucleoplasm and the nucleolus, whereas RipAS is only found in the nucleoplasm. It is unlikely that RipAS can interact with StTOPP6 within the nucleolus. Moreover, with the re-localization of the RipAS from the nucleoplasm, the fluorescence of StTOPP6 was unperturbed (Fig. 4B). Taken together, in the absence of detectable changes in expression, the observed reduction of StTOPP6 in the nucleolus may be due to that RipAS inhibits StTOPP6 from localizing to the nucleolus. Critically, StTOPP6 nucleolar fluorescence is also reduced in OE-StTOPP6 lines inoculated with UW551, rather than inoculated with *ripAS* deletion mutant, we propose that the function of RipAS is unique in T3Es.

RipAS has been shown to interact with StTOPP6, causing reduced accumulation of StTOPP6 in nucleolus. From the current results, RipAS conforms to the characteristics of PP1-interacting proteins (PIPs). The subcellular localization and the substrate specificity of PP1 catalytic subunits are determined by PIPs, which can localize PP1 to distinct regions of the cell and modulate its substrate specificity [30–32]. Most PIPs bind PP1 through a primary PP1-binding motif, a so-called ‘RVXF’ sequence, which generally conforms to the consensus sequence [K/R][K/R][V/I][x][F/W] [33]. PIPs function as targeting subunits, substrates, and/or inhibitors. Furthermore, there is a pathogenic virulence factor has been reported as PP1 regulatory subunits. A *P. infestans* effector Pi04314 as a PIP and causes PP1 catalytic subunit re-localization from the host nucleolus [24]. A similar phenomenon showed between RipAS and StTOPP6 and we guess that the T3Es RipAS is also a PP1-interacting protein, just like the effector Pi04314. However, the difference is that RipAS does not contain an RVxF motif, so we cannot make further confirmation of whether it is PIP. Our future efforts will focus on the impact of the reduced nucleolar accumulation of StTOPP6 to better understand the mechanisms by which effector RipAS alters host processes.

Six PP1 family members were identified in potato (termed StPP1–1, StPP1–2, StPP1–3, StPP1–4, StPP1–5, and StTOPP6). The StPP1–4, StPP1–5, and StTOPP6 correspond to previously reported StPP1c-1/2/3, respectively [24]. The same results were shown, the other PP1 proteins not only interacted with RipAS but also showed reduced accumulation of StTOPP6 in nucleolus (Fig. 5A and B). This predicts that RipAS may target the PP1 family. RipAS was conserved in *R.solanacearum* species complex; RipAS of other strains can also reduce the nucleolar accumulation of StTOPP6 (Fig. 6). Because RipAS and PP1s are highly conserved, we hypothesized that the association between RipAS and PP1 proteins may widely exist in the interaction between *R. solanacearum* and hosts. This virulence strategy may be a case of convergent evolution between *R. solanacearum* and hosts. In conclusion, we predict that RipAS target type one protein phosphatases to promote bacterial wilt. Our study suggests that RipAS is a key virulence factor during *R. solanacearum* infection.

## Materials and methods

### Plant and bacterial materials

*R. solanacearum* strains were incubated on BG medium (bactopeptone 10 g/L, glucose 2.5 g/L, and casino acids 1 g/L) under 28°C for 60 h.

*Solanum tuberosum* E3 and *Nicotiana benthamiana* plants were grown at 20°C in a climate chamber with cycles of 16/8 h day/night. Three-week-old potato plants were used for the virulence identification and five-week-old *N. benthamiana* was used for transient expression. For bacterial quantification, four potato plants were cultured on Murashige-Skoog (MS) medium with 3% (w/v) sucrose and kept at 22°C under a photoperiod of 16/8 h day/night with light intensities ranging from 400 to 1000 μmol m^−2^ s^−1^. For tuberization tests, 3-week-old potato in climate chambers were transplanted into plastic pots in net houses. After 12 weeks, tubers were harvested and fresh weight per plant was quantified.

### Yeast two-hybrid (Y2H) assays

The full-length coding sequences were cloned into the pGADT7 vector or the pGBKT7 vector. The primers of related genes were listed in Table S1 (see online supplementary material). Target combinations were transformed into AH109 yeast and incubated on selective SD/−Leu/−Trp/-His/−Ade medium with Aureobasidin A (AbA) and X-α-gal.

### Immunoblot analysis and co-immunoprecipitation (co-IP)

The Immunoblot and Co-IP assays were performed in *N. benthamiana* as described [34]. *N. benthamiana* leaves expressing the target proteins by transient expression were collected at 2 d post-infection. The full length of StTOPP6/m was amplified and cloned into pH7C with FLAG/GFP tag, while the RipAS was amplified and cloned into vector pH7C with HA tag.

### Mutagenesis of *R. solanacearum*

The Δ*ripAS* deleting mutant and *ripAS* complementation strains were generated as described previously [29]. The whole *ripAS* gene was replaced with *Km*^r^ [35] in WT UW551 to generate the Δ*ripAS* deleting mutant. The *ripAS* complementation strain was constructed by inserting *ripAS* and *Sm^r^* with native promoters in a permissive chromosomal site of Δ*ripAS* mutant [36].

### Vector construction and potato transformation

The StTOPP6 and RipAS were driven by 35S promoters to generate overexpressing transgenic potato. The full-length coding sequences were cloned into vector pBI121 with GFP tag. The constructs were transformed into E3 potato, as previously described [37]. The StTOPP6-overexpressing plants were identified by qRT-PCR, while the RipAS-overexpressing lines were identified by semiquantitative PCR.

### Pathogenicity assays

A total of 48 (6 × 8) plants in a pot were inoculated with 500 mL (OD_600_ = 0.1) *R. solanacearum* suspension by soil drench. The surviving plants were recorded daily after inoculation to analyse the survival rates, three independent experiments were evaluated. Concerning the resistance of the transgenic plants, six plants were used for the investigation of the Area under Disease Progress Curve (AUDPC), as described previously [38].

*In vitro* potato tests were used for bacterial quantification and inoculated with 5.0 ml of *R. solanacearum* suspension (OD_600_ = 0.1). The middle of potato roots five days after inoculation was harvested and weighed in a sterile environment. Root samples were ground in 5 ml of sterile water, and dilution plated onto BG medium. After incubating 48 h at 28°C, colonies were counted [25].

### RNA extraction, qRT-PCR, and RNA-Seq

Total RNA was extracted using a Total RNA Kit (ZOMANBIO, ZP405). The programs of the qRT-PCR were 95°C for 15 min, followed by 40 cycles of 95°C for 15 s, 60°C for 30 s, and 72°C for 30 s. The potato gene *Ef1a* (XM_006343394) was used as a control gene to normalize the expression data [39]. All primer sequences for qRT-PCR analysis are described in Table S2 (see online supplementary material). For RNA-Seq analysis, roots of 3-week-old (E3, OE-RipAS-6, and OE-RipAS-10) plants grown *in vitro* were sampled for RNA-seq, as described previously [29].

### Confocal laser scanning microscopy

The target sequences were cloned into the pK7WGF2 vector with the GFP fluorescence tag or the pK7WGF2 vector with the RFP fluorescence tag. The confocal images were captured with a Leica SP8 (Leica, Wetzlar, Germany) instrument. Images were recorded in two channels: GFP (excitation wavelength 488 nm, emission filter 500–525 nm) and RFP (excitation wavelength 552 nm, emission filter 580–620 nm).

## Acknowledgments

We thank Dr Caitilyn Allen and the late Dr Philippe Prior for providing us with *R. solanacearum* strains. We also thank all members of the potato group in Huazhong Agricultural University for supporting this project. This work was funded by the National Natural Science Foundation of China (32201789) and the China Agriculture Research System of MOF and MARA (CARS-09).

## Author contributions

Conceived and designed the experiments: B.W., B.S., H.C. Performed the experiments: B.W., W.H., M.H., Y.W., Y.L., C.M. Analysed the data: B.W., W.H., L.Y., D.Z., D.C. Prepared the manuscript: B.W., M.H., B.S., H.C. Revised the manuscript: B.W., M.H., J.F., C.M., D.Z., D.C., N.T., B.S., H.C.

## Data availability

All data supporting the findings of this research are available within the paper and within its supplementary data published online.

## Conflict of interest

The authors declare no competing interests.

## Supplementary data

Supplementary data is available at *Horticulture Research* online.

## Supplementary Material

Web_Material_uhad087
