# Supplementary material for: Ralstonia solanacearum type III effector RipAS associates with potato type one protein phosphatase StTOPP6 to promote bacterial wilt
Source: Hortic Res. 2023 May 3;10(6):uhad087. doi: 10.1093/hr/uhad087 (PMC10273071; doi:10.1093/hr/uhad087)
Supplement: Web_Material_uhad087 [file web_material_uhad087.zip › Supporting information.docx]

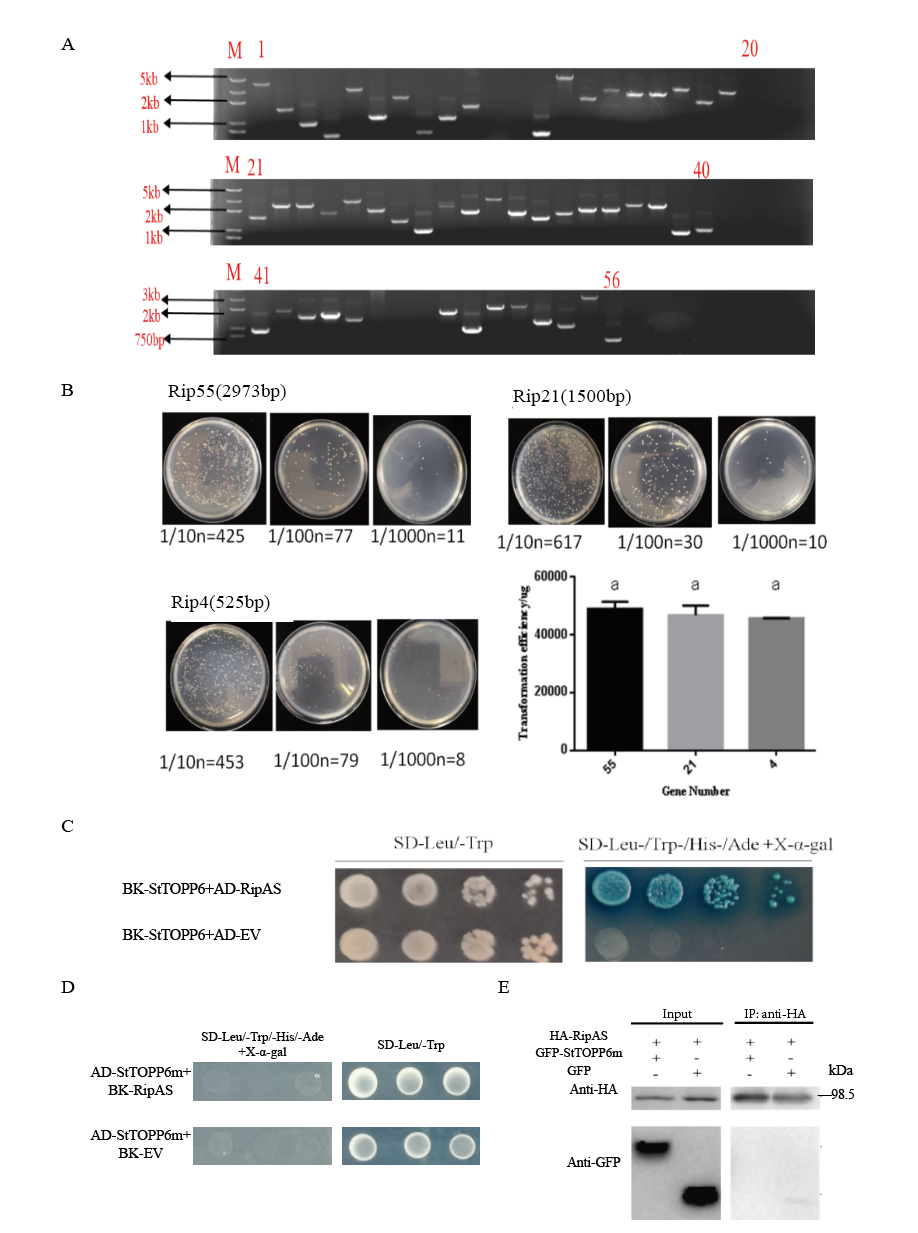
**Supporting Information**

**Figure S1. The construction and screening of UW551 effectors yeast cDNA library**

A. Gel electrophoresis detection of 52 effectors prey vector. B. The plates growth condition and a bar chart of efficiency about three effector prey vectors transformed BK-StTOPP6 decoy bacteria to calculate efficiency. C. Target protein RipAS and control AD plasmid were transformed yeast competent cell which contained BK-StTOPP6. Then, blue verification was performed on DDO, QDO+X-α-gal plates. D. Yeast two-hybrid (Y2H) assay for interactions between RipAS and StTOPP6m. E. RipAS does not interact with StTOPP6m in the Co-IP assay.


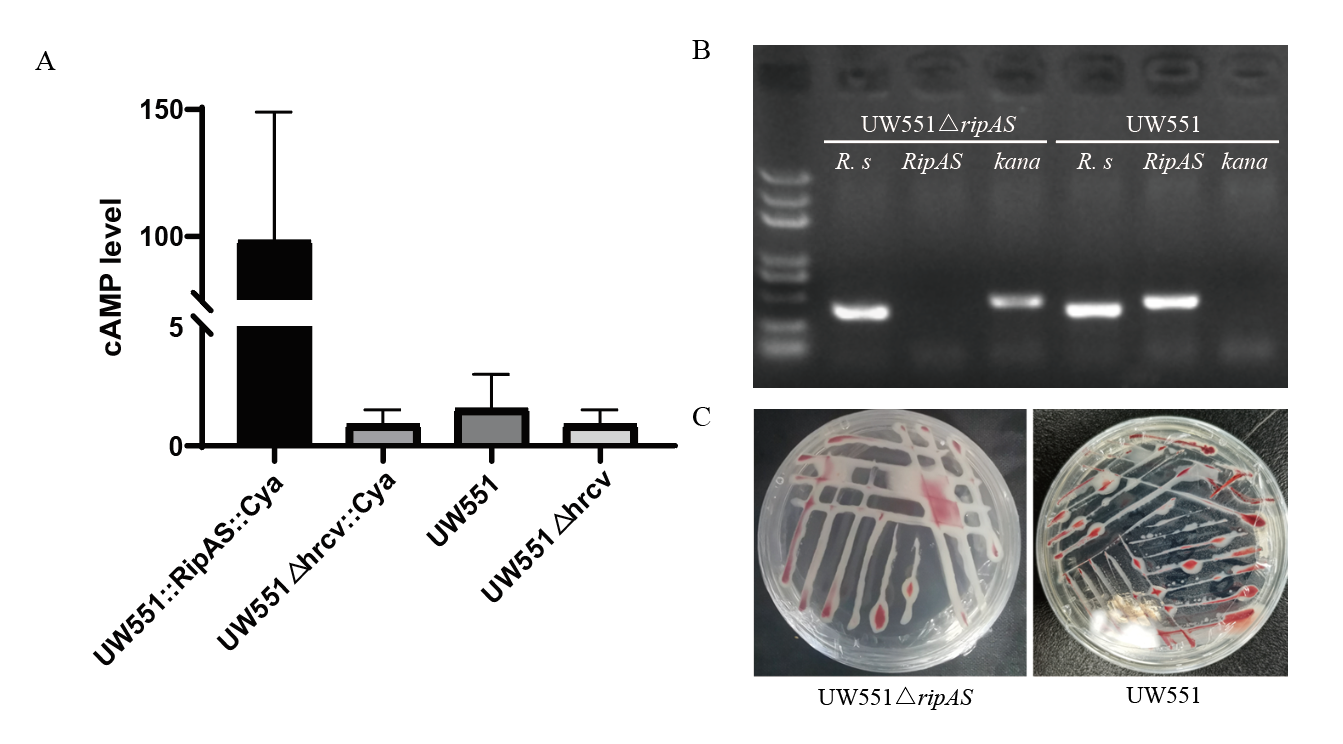
**Figure S2. The construct of ripAS muntant of *R. solanacearum* UW551.**

A. RipAB is a type III-secreted effector. The cyclic adenosine monophosphate (cAMP) levels were measured at 7 days post-inoculation (dpi). B. The presence of the gene *ripAS* in the wild-type UW551 and the UW551Δ*ripAS* mutant. C. Morphological of the UW551 and the UW551Δ*ripAS* mutant strains.


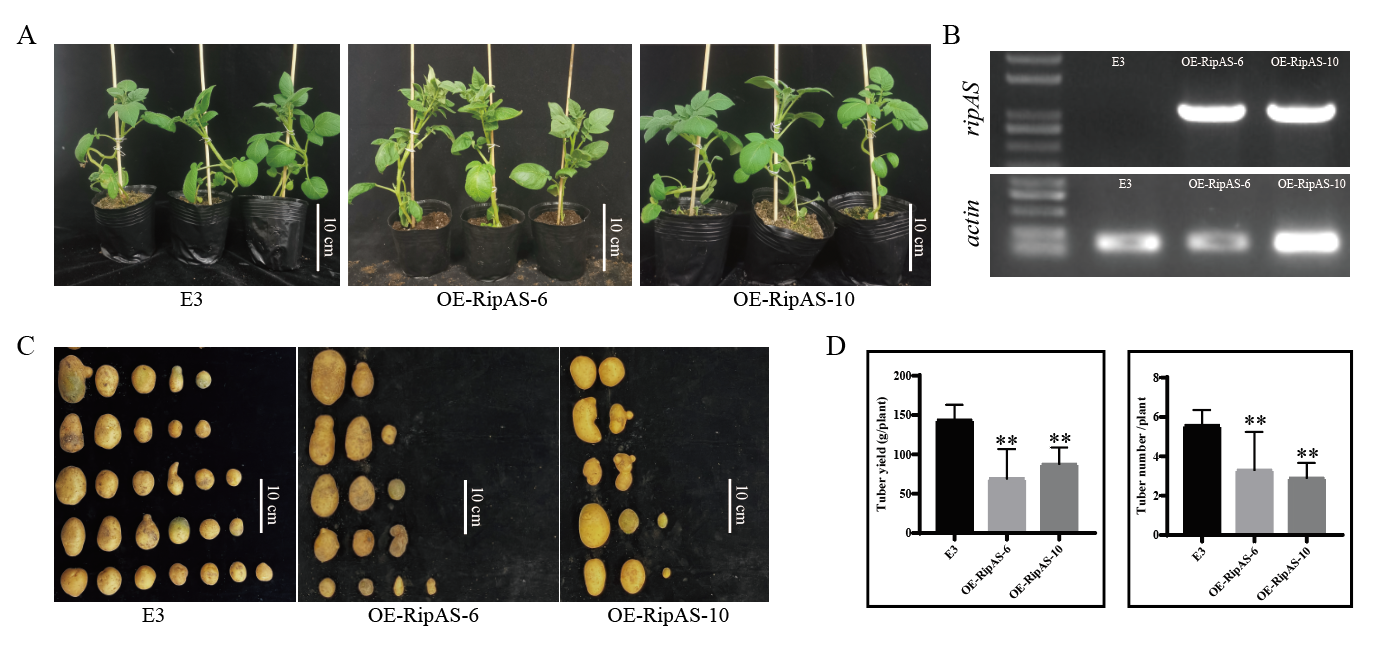


**Figure S3. The phenotype of OE-RipAS lines.**

A, Representative photos of Ri-StTOPP6 plants and WT E3 plants grown in pots for 14 days in the greenhouse. B. Semi-quantitative polymerase chain reaction (PCR) of regenerated RipAS transgenic potato plants. C-D. Tuber yield and numbers for soil-grown E3 and OE-RipAS transgenic plants. Data were obtained at 70 days in the net house with ten plants for each genotype. Data are presented as mean± standard deviation, n=10. The asterisks indicate a statistically significant difference (Student’s t test, **P <0.01).


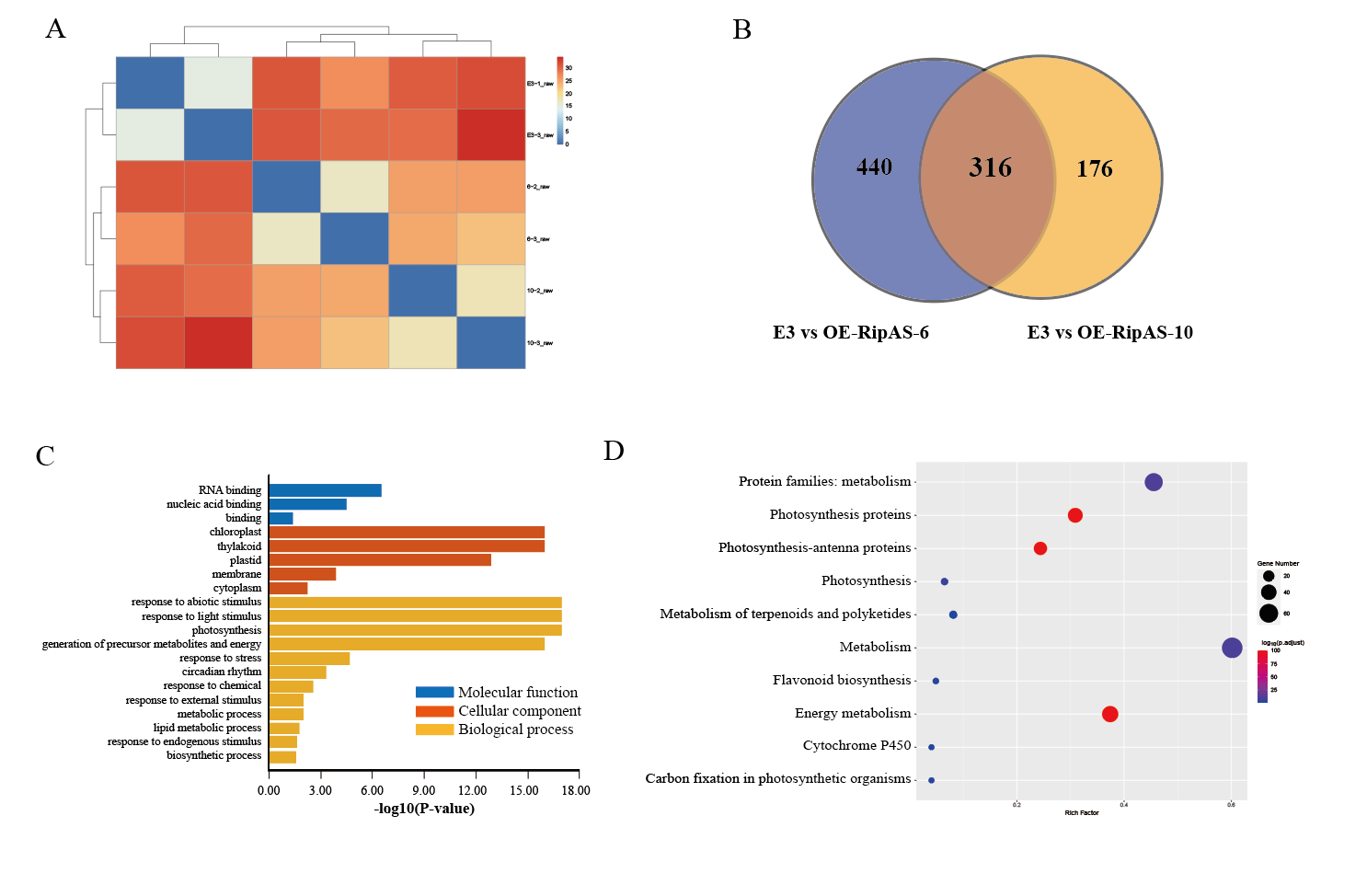


**Figure S4. RNA-seq analysis of OE-RipAS lines**

A. Clustering result of OE-RipAS transgenics lines and E3 shown as a heatmap. B. Venn diagram showing the DEGs in OE-RipAS transgenics lines compared to the control plant E3. C. Gene Ontology enrichment analysis of DEGs in OE-RipAS transgenics lines. *P*‐values (FDR) <0.05. D. KEGG enrichment analysis of DEGs in OE-RipAS lines. *P*‐values (FDR) <0.05


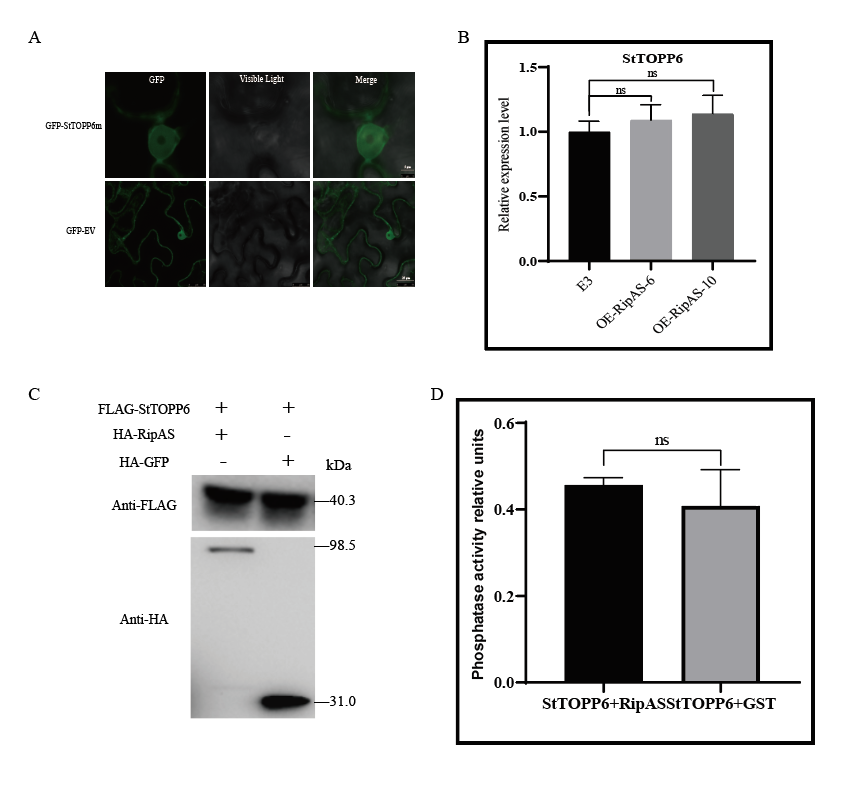


**Figure S5. RipAS does not affect the expression of StTOPP6.**

1. StTOPP6m absent from the nucleolus. Scale bar is 5 µm. B. The expression level of StTOPP6 is not disturbed in OE-RipAS lines. The expression of StTOPP6 in the control plants was set to ‘1’. Experiments were repeated three times, with three independent biological repetitions each time. Error bars indicate the standard error. Relative transcript levels were normalized to the transcript levels of *Stef1α*. C. The accumulation of StTOPP6 is stable when co-expressed with RipAS. Size markers are in kDa. D. The phosphatase activity of StTOPP6 is not affected by the RipAS. Data represent means ± standard deviation, n=3. The “ns” indicate no significant difference (Student’s t test,(ns)*P* >0.05).


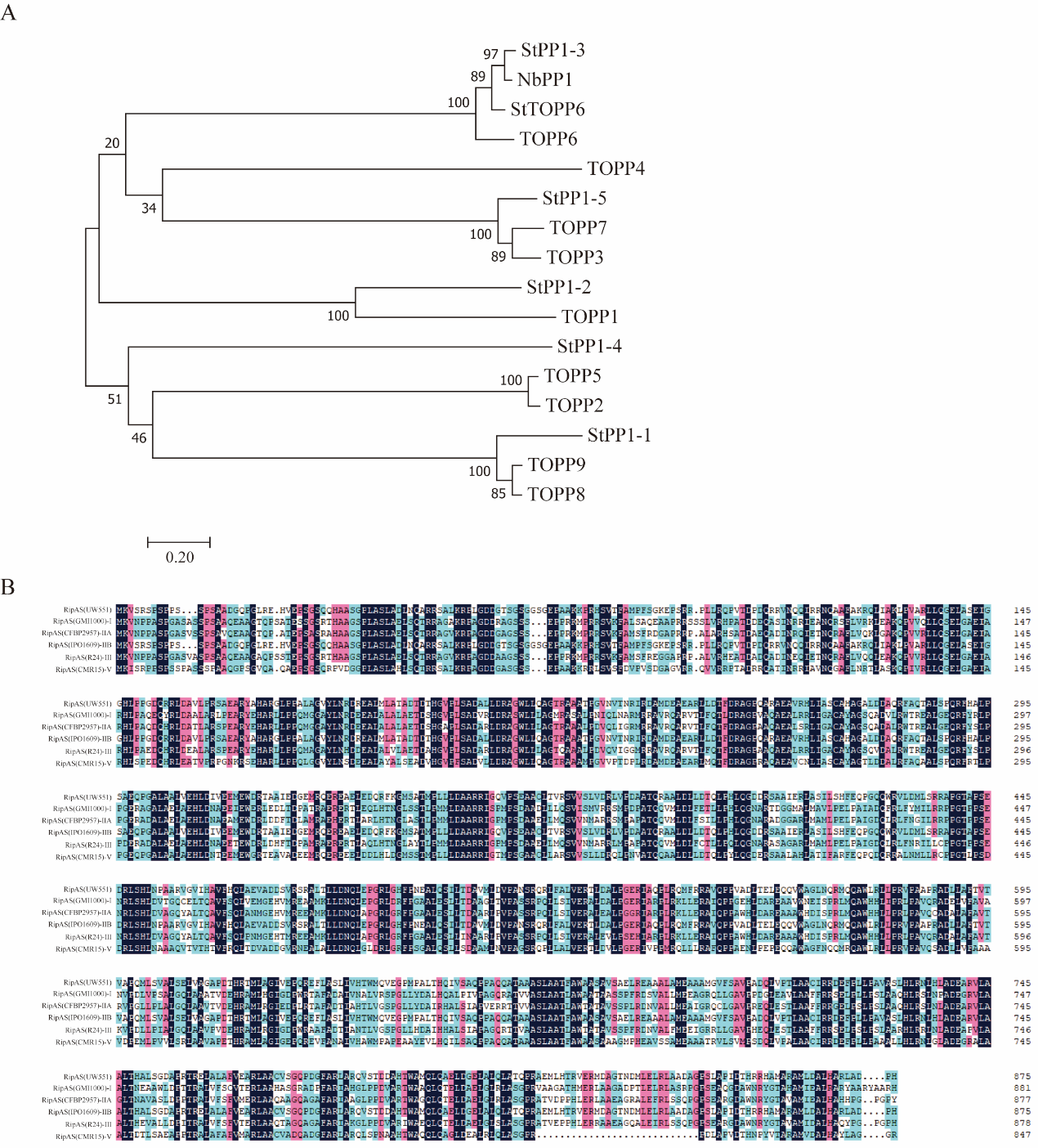


**Figure S6. The amino acid alignment of PP1s and RipAS sequences**

1. Phylogenetic tree of PP1s sequences from Arabidopsis, N. benthamiana, and *S. tuberosum.* The resulting tree was rendered with MEGA. StPP1-4, StPP1-5, and StTOPP6 corresponds to the StPP1c-1/2-3^24^. B*.* Alignment of RipAS sequence from different phylotype *R. solanacearum* strains.

Table S1. List of cloning primers used in this study.

| **Primer name** | **Sequence (5’-3’)** | **Target gene** | **Usage** |
| --- | --- | --- | --- |
| BK-RipAS-EcoRI-F | gcatatggccatggaggccgaattcATGAAAGTCAGCCGCTCACC | RRSL_00571 | Y2H assay |
| BK-RipAS-BamHI-R | cggccgctgcaggtcgacggatccTCAATCATGCGGGTCGGC | RRSL_00571 | Y2H assay |
| AD-StTOPP6/m-EcoRI-F | TGGCCATGGAGGCCAGTGAATTCATGGACCAGAATGTGTTGGAT | Soltu.DM.05G025610.1 | Y2H assay |
| AD-StTOPP6/m-BamHI-R | AGCTCGAGCTCGATGGATCCTCATGCTTTGGAATTAAAAAAGGA | Soltu.DM.05G025610.1 | Y2H assay |
| HA-RipAS-StuⅠ-F | tggacgagctgtacaagATGAAAGTCAGCCGCTCACC | RRSL_00571 | Co-IP |
| HA-RipAS-StuⅠ-R | gatatcaccactttgtacaTCAATCATGCGGGTCGGC | RRSL_00571 | Co-IP |
| GFP-StTOPP6/m-StuⅠ-F | acgccgagATGGACCAGAATGTGTTGGATG | Soltu.DM.05G025610.1 | Co-IP |
| GFP-StTOPP6/m-StuⅠ-F | gggaagagTCATGCTTTGGAATTAAAAAAGGAC | Soltu.DM.05G025610.1 | Co-IP |
| FLAG- StTOPP6-StuⅠ-F | acgccgagATGGACCAGAATGTGTTGGATG | Soltu.DM.05G025610.1 | Co-IP |
| FLAG- StTOPP6-StuⅠ-R | gggaagagTCATGCTTTGGAATTAAAAAAGGAC | Soltu.DM.05G025610.1 | Co-IP |
| Kana-RipAS-F | ATGAAAGTCAGCCGCTCGTTTCAAAATCGGCTCCGTCGATAC | RRSL_00571 | Mutagenesis |
| Kana-RipAS-R | CCATCAATCATGCGGGTCGGCAAGCCCTGCACGAATACCAGC | RRSL_00571 | Mutagenesis |
| OE-RipAS-F | tggacgagctgtacaagATGAAAGTCAGCCGCTCACC | RRSL_00571 | Overexpression |
| OE-RipAS-R | gatatcaccactttgtacaTCAATCATGCGGGTCGGC | RRSL_00571 | Overexpression |
| OE-GFP-StPP1-F | tggacgagctgtacaagATGGACCAGAATGTGTTGGATG | Soltu.DM.05G025610.1 | Overexpression |
| OE-GFP-StPP1-R | gatatcaccactttgtacaTCATGCTTTGGAATTAAAAAAGGAC | Soltu.DM.05G025610.1 | Overexpression |
| RFP-RipAS- Bsp1407Ⅰ-F | cggcgccgatatcacaagtttgtacATGAAAGTCAGCCGCTCACC | RRSL_00571 | Subcellular localization |
| RFP-RipAS- Bsp1407Ⅰ-R | gccgcgggatatcaccactttgtacTCAATCATGCGGGTCGGC | RRSL_00571 | Subcellular localization |
| GFP-PP1(m)- Bsp1407Ⅰ-F | tggacgagctgtacaagATGGACCAGAATGTGTTGGATG | Soltu.DM.05G025610.1 | Subcellular localization |
| GFP-PP1(m)- Bsp1407Ⅰ-R | CGGGATATCaccactttgtacaTCATGCTTTGGAATTAAAAAAGGA | Soltu.DM.05G025610.1 | Subcellular localization |
| AD-StPP1-1-EcoRI-F | TGGCCATGGAGGCCAGTGAATTCATGTCGAGGAAGAAG | XM_006354429.2 | Y2H assay |
| AD-StPP1-1-BamHI-R | AGCTCGAGCTCGATGGATCCTCATCCCATTTTACCCAGAAATGA | XM_006354429.2 | Y2H assay |
| AD-StPP1-2-EcoRI-F | TGGCCATGGAGGCCAGTGAATTCATGGCACAAAATGAGCATCAGC | XM_006358645.2 | Y2H assay |
| AD-StPP1-2-BamHI-R | AGCTCGAGCTCGATGGATCCTCATAAGAACCGAGGTTTTCTATCTG | XM_006358645.2 | Y2H assay |
| AD-StPP1-3-EcoRI-F | TGGCCATGGAGGCCAGTGAATTCATGGATATTTTAGCACTTGATG | XM_006350625.2 | Y2H assay |
| AD-StPP1-3-BamHI-R | AGCTCGAGCTCGATGGATCCTTACCAAGAAATGACCTTCGTTTTTG | XM_006350625.2 | Y2H assay |
| AD-StPP1-4-EcoRI-F | TGGCCATGGAGGCCAGTGAATTCATGGCTCAAAATGGGCAGG | XM_006350729.2 | Y2H assay |
| AD-StPP1-4-BamHI-R | AGCTCGAGCTCGATGGATCCTCACAAGAACCGAGGTTTTCTATC | XM_006350729.2 | Y2H assay |
| AD-StPP1-5-EcoRI-F | TGGCCATGGAGGCCAGTGAATTCATGGACCCTGCAGCTG | XM_006346756.2 | Y2H assay |
| AD-StPP1-5-BamHI-R | AGCTCGAGCTCGATGGATCCTCACATCATAAACTTATTTTTCTTC | XM_006346756.2 | Y2H assay |
| AD-TOPP1-EcoRI-F | TGGCCATGGAGGCCAGTGAATTCATGGCGGAGAAGCCGG | AT2G29400 | Y2H assay |
| AD-TOPP1-BamHI-R | AGCTCGAGCTCGATGGATCCTCACAGAAAAGGCGACTTCTTTTC | AT2G29400 | Y2H assay |
| AD-NbPP1-EcoRI-F | TGGCCATGGAGGCCAGTGAATTCATGGATTCTTTAGCACTTGATG | Niben101Scf03064g08007.1 | Y2H assay |
| AD-NbPP1-BamHI-R | AGCTCGAGCTCGATGGATCCTCATCCAACTTTACCAAGAAATGAC | Niben101Scf03064g08007.1 | Y2H assay |
| BK-RipAS(GMI1000)-F | atggccatggaggccgaattcATGAAAGTCAATCCACCCGCT | RSp1384 | Y2H assay |
| BK-RipAS(GMI1000)-R | cggccgctgcaggtcgacggatccTCAAGCGTACGGGCCG | RSp1384 | Y2H assay |
| BK-RipAS(CFBP2957)-F | AtggccatggaggccgaattcATGAAAGTCAACCCTCCCGC | RCFBP_mp30150 | Y2H assay |
| BK-RipAS(CFBP2957)-R | cggccgctgcaggtcgacggatccTCAAGCGTGCCGGGC | RCFBP_mp30150 | Y2H assay |
| GFP-StPP1-1-Bsp1407Ⅰ-F | acgccgagATGGACCAGAATGTCGAGGAAGAAG | XM_006354429.2 | Subcellular localization |
| GFP-StPP1-1-Bsp1407Ⅰ-R | CGGGATATCaccactttgtacaTCATCCCATTTTACCCAGAAATGA | XM_006354429.2 | Subcellular localization |
| GFP-StPP1-2-Bsp1407Ⅰ-F | acgccgagATGGACCAGAATGGCACAAAATGAGCATCAGC | XM_006358645.2 | Subcellular localization |
| GFP-StPP1-2-Bsp1407Ⅰ-R | CGGGATATCaccactttgtacaTCATAAGAACCGAGGTTTTCTATC | XM_006358645.2 | Subcellular localization |
| GFP-StPP1-3-Bsp1407Ⅰ-F | acgccgagATGGACCAGAATGGATATTTTAGCACTTGATG | XM_006350625.2 | Subcellular localization |
| GFP-StPP1-3-Bsp1407Ⅰ-R | CGGGATATCaccactttgtacaTTACCAAGAAATGACCTTCGTTTT | XM_006350625.2 | Subcellular localization |
| GFP-StPP1-4-Bsp1407Ⅰ-F | acgccgagATGGACCAGAATGGCTCAAAATGGGCAGG | XM_006350729.2 | Subcellular localization |
| GFP-StPP1-4-Bsp1407Ⅰ-R | CGGGATATCaccactttgtacaTCACAAGAACCGAGGTTTTCTATC | XM_006350729.2 | Subcellular localization |
| GFP-StPP1-5-Bsp1407Ⅰ-F | acgccgagATGGACCAGAATGGACCCTGCAGCTG | XM_006346756.2 | Subcellular localization |
| GFP-StPP1-5-Bsp1407Ⅰ-R | CGGGATATCaccactttgtacaTCACATCATAAACTTATTTTTCTTC | XM_006346756.2 | Subcellular localization |
| GFP-TOPP1-Bsp1407Ⅰ-F | acgccgagATGGACCAGAATGGCGGAGAAGCCGG | AT2G29400 | Subcellular localization |
| GFP-TOPP1-Bsp1407Ⅰ-R | CGGGATATCaccactttgtacaTCACAGAAAAGGCGACTTCTTTTC | AT2G29400 | Subcellular localization |
| GFP-NbPP1-Bsp1407Ⅰ-F | acgccgagATGGACCAGAATGGATTCTTTAGCACTTGATG | Niben101Scf03064g08007.1 | Subcellular localization |
| GFP-NbPP1-Bsp1407Ⅰ-R | CGGGATATCaccactttgtacaTCATCCAACTTTACCAAGAAATG | Niben101Scf03064g08007.1 | Subcellular localization |
| RFP-RipAS(GMI1000)-F | cggcgccgatatcacaagtttgtacATGAAAGTCAATCCACCCGCT | RSp1384 | Subcellular localization |
| RFP-RipAS(GMI1000)-R | gccgcgggatatcaccactttgtacTCAAGCGTACGGGCCG | RSp1384 | Subcellular localization |
| RFP-RipAS(CFBP2957)-F | cggcgccgatatcacaagtttgtacATGAAAGTCAACCCTCCCGC | RCFBP_mp30150 | Subcellular localization |
| RFP-RipAS(CFBP2957)-R | gccgcgggatatcaccactttgtacTCAAGCGTGCCGGGC | RCFBP_mp30150 | Subcellular localization |

Table S2. List of qRT-PCR primers used in this study.

| Primer name | Sequence (5’-3’) | Target gene |
| --- | --- | --- |
| StTOPP6-q-F | TGAGTATGGTGGATTGCCGC | Soltu.DM.05G025610.1 |
| StTOPP6-q-R | ACATTCCGCATTTCCCCGAA | Soltu.DM.05G025610.1 |
| RipAS-q-F | TCCTCAGCCATTTCGAGCAG | RRSL_00571 |
| RipAS-q-R | CCAACTGGTGAAAAACCGCA | RRSL_00571 |
| Stef1α-q-R | ATTGGAAACGGATATGCTCCA | Soltu.DM.06G005620.1 |
| Stef1α-q-R | TCCTTACCTGAACGCCTGTCA | Soltu.DM.06G005620.1 |
